# Estimating complete migration probabilities from grouped data: A methods protocol for developing a global Human Internal Migration Database

**DOI:** 10.1371/journal.pone.0315389

**Published:** 2024-12-10

**Authors:** Sigurd Dyrting, Andrew Taylor

**Affiliations:** Northern Institute, Charles Darwin University, Darwin, Northern Territory, Australia; Universidade Federal de Minas Gerais, BRAZIL

## Abstract

The majority of migration moves globally are internal within national borders. This makes internal migration intensities an important component for understanding the dynamics of population change according to size, composition and across geographies. While incorporating migration into demography’s quantitative framework allows a description of population change across both time and space, and mathematical and conceptual frameworks for migration have been developed, researchers lack a public repository of historical age-origin-destination-specific migration probabilities that is in a common format and spans a range of countries. Addressing this requires a robust method for inferring migration probabilities from census and survey data when there are significant levels of uncertainty from small-sample noise and age aggregation. In this paper we extend the P-TOPALS and P-spline methods for smoothing migration probabilities to apply to grouped data by ages to develop a methods protocol for a harmonised, homogeneous format and multi-nation Human Internal Migration Database. We find our method out-performs a hybrid spline-parametric method in terms of both accuracy and plausibility. We illustrate the method by estimating complete age-origin-destination migration probabilities for more than 50 countries using microdata samples from IPUMS International. This work advances the stock of migration data from which demographers and others can draw from in the analysis and projection of population change.

## Introduction

The majority of people who migrate move within their own country rather than internationally [1, 2]. Internal migration probabilities, along with fertility and mortality rates, are the primary processes for understanding and describing the dynamics of spatial changes in the sizes and structures of sub-national populations, especially for countries that have completed the first demographic transition, since that process leads to a convergence towards lower vital rates [3], although significant intra and inter-national variations in fertility and mortality rates do remain, including, for example, mortality rates within the United States [4]. Because of this, internal migration has become the key driver of population change and having a complete picture of migration probabilities is important for their application to constructing sub-national multiregional life tables [5], stable age structures [6], subnational population projections [7, 8], and measures of the intensity of migration and its effectiveness in redistributing population [3, 9, 10]. They are also increasingly a focus for government policies and initiatives for population attraction in countries and sub-national jurisdictions experiencing reduced natural growth rates and increasing rates of urbanization and regional depopulation [11, 12]. In this article migrations are internal unless stated otherwise.

The development of a theoretical framework for migration began with Ravenstein’s observation in the late 1800s that human movement was patterned [13, 14]. The literature on migration evolved to characterise its diffusive nature as being a balance between new opportunities for the individual at the destination and intervening ones at the origin [15], with differences in actual and perceived positive and negative factors at the origin and destination, tempered by intervening obstacles [16]. Migration has subsequently been described as being selective by demographic and socioeconomic characteristics [17, 18], and part of a mobility transition progressing sequentially in time and radiating in space as a society passes through modernisation [19]. Empirical studies have continued to document the evolution of migration in European countries and their former colonies [20–24] and beyond [3, 25, 26].

Incorporating migration into demography’s quantitative framework allows a description of population change in size and composition across both time and space. In this article we work within the multistate demography framework [27–29]. In this approach, the fundamental variable is a complete schedule of migration rates, a matrix of probabilities that a person at place *O* (the origin) with age *x* will be at place *D* (a potential destination) with age *x* + *n* an interval *n* years later. The labels *O* and *D* refer to a given spatial decomposition and age *x* ranges by single year increments from birth to the limit of human life ω. The triplet (*O*,*D*,*x*) therefore labels the three dimensions of a migration ’cube’ [30]. As well as allowing the fore mentioned calculations at high age resolution, robust methods for estimating complete migration probability schedules are a necessary precursor to resolving one of the main problems frustrating spatial demography and subsequently its application to infrastructure and service planning: data. This is because, in migration studies, mathematical and conceptual frameworks are more advanced than the available data [31] and, while for vital processes public repositories of historical data exist [32, 33], for internal migration there are no such repostitories for the demographer to draw from.

The works of P. Rees & Kupiszewski [30] and Bell et al. [34] show that data problems for migration take a number of forms. First, most data on internal migration are collected periodically rather than continuously, either by population censuses or surveys, and usually every 10 or 5 years. This impacts the quality of the estimated migration probabilities by preventing the pooling of counts over consecutive years. Secondly, there are significant variations in the types of migration data collected between countries, with population registers capturing changes in address (migration events) and population censuses and surveys recording addresses at two or more points in time usually separated by a fixed duration (migration transitions) [35, 36]. Even for transition-style data there are variations in the interval *n*, with Bell et al. [34] finding that, of the 142 countries they surveyed that collected internal migration data in the 2000 UN census round, 29 (20%) collected 1-year data, 52 (37%) collected 5-year data, and 32 (23%) collected data over some other interval. The enduring and ongoing nature of migration as a demographic event means that transition data over long intervals will underestimate the number of moves, making migration probabilities over different intervals not directly comparable [36, 37].

Lastly, internal migration data is mostly compiled and published by national statistics offices based on varying sources, methods and formats, making international comparative demographic studies on internal probabilities labour intensive according to the number of countries in the study. The work required includes obtaining access permission, data extraction, and potentially significant fees for bespoke data requests for tables which are not made publicly available [30, 38]. Additionally, published data is often incomplete, such that it is not possible to derive accurate migration probabilities for all combinations of origins, destinations, and ages, either because data is missing or because sample-based estimates of probabilities are noisy with high standard errors. The problem of incomplete data is particularly prevalent for internal migration because one cannot avoid estimating quantities at the subnational level.

Converting migration data to complete schedules of internal migration probabilities would mitigate many of these problems, and free the user from issues of data type (transition or event) by harmonising into one common data structure. This is relevant for subnational population projections where age groups must map into each other over time as individual age groups get one increment older, and also facilitates the fitting of model migration schedules, which are more highly parameterised than mortality and fertility models due to the presence of multiple peaks [39]. Complete migration probability schedules also allow a wider dissemination of migration data because estimates of migration probabilities that account for finite sample noise are less disclosive than the data they are derived from, being in effect a data treatment technique for aggregated data [40], alleviating potential ethical issues around the identification of individuals or indeed minority groups.

In the case of transition-style migration measures, the data from which migration probabilities are derived are census or survey origin-destination matrices by sex and age. In general, the age variable is grouped. That is, some or all values represent an age range spanning an interval greater than one year. It is difficult to assess the extent of grouped migration data because, as both P. Rees & Kupiszewski [30] and Bell et al. [38] stress in their review of national statistical offices, there can be considerable variance between the data that is collected (available ’in principle’) and data that is published (available ’off the shelf’). If we consider off-the-shelf census data provided by national statistics offices from the group of six countries listed in Bell et al. [38, Census Catalogue section] as recording changes in address over two intervals (Australia, 2011; Canada, 2001; Malta, 2005; Greece, 2001; Portugal, 2001; South Korea, 2000), and confine ourselves to first subnational geographic levels, we find that Australia provides access to origin-destination tables by single-year of age [41], Canada provides origin-destination tables without age disaggregation and tables of out- and in-migration by five-year age groups [42], Malta provides tables by ten-year age groups [43], Greece by ten-year age groups [44], the tables provided by Portugal are not disaggregated by age [45], and South Korea provides counts of out- and in- migrants by five-year age groups [46]. Of the five countries that publish data disaggregated by age, four used aggregated age groups. A solution to this data problem in spatial demography will therefore require a method for inferring migration probabilities from age-specific origin-destination matrices that can be applied to both single-year and age-grouped data.

Current methods for inferring single-year of age migration probabilities for grouped data are based on the property that grouped probabilities are a weighted average where the weights are proportional to the single-year population [47]. Spline methods seek to construct a complete curve that reproduces the grouped probabilities. If the weights do not vary appreciably over each age group, then we can approximate them by equal weights. This approximation was used by Campbell [48] for the case of five-year age groups to interpolate a complete curve from grouped data using Sprague multipliers [49], and by Rogers et al. [47] to interpolate out-migration from Stockholm using cubic splines [50]. If it is further assumed that probabilities are approximately linear in age over the group interval, then the grouped probability is approximately equal to the probability at the mid point of the age group. This approximation was use in Rogers et al. [51] as a basis for cubic spline interpolation.

Internal migration probabilities have a common profile over the life course: decreasing with age for children, then increasing for young adults as they move for further education or careers, reaching a maximum in the twenties, thereafter declining with age, with possibly a secondary increase at ages associated with peaks in retirement or post-retirement moves [18]. Model migration schedules describe this pattern using a specific functional form for each component, the magnitude, position, and width of which is explained by a set of parameters [39, 47, 52–54]. One of the first applications of model migration schedules was in the expansion of grouped data [47], where population weights were used to estimate one-year rates from five-year age data. Rogers & Castro [55] advocated the use of the mid-point approximation for fitting the model parameters, and Rogers et al. [51] used a hybrid approach, interpolating a compete curve using cubic splines and the mid-point approximation, and then fitting the result with a model migration schedule.

While methods based on splines and model migration schedules have their advantages, they both have limitations that make them unsuitable as general frameworks for estimating migration probabilities from grouped data. Spline methods are easy to calibrate and have great flexibility in the age profiles they can fit because they only assume the profile is locally polynomial. However, they make assumptions about the population distribution or migration probability over each age group which are not likely to hold as the length of the group interval increases. For example, when the age groups are terminated by an open interval such as 80 years and over, the population weights will exponentially decrease rather than remain constant, and for age groups spanning a local maxima such as a student or labour peak, migration probabilities will display significant convexity rather than change linearly with age. Spline methods also assume observations are free from significant sample noise which limits their application to large populations and age groupings greater than one year. Model migration schedules have the advantage that, when properly calibrated, they will produce complete schedules that reflect the features their parameters encode, but their accuracy is constrained by the assumed functional form. As a result, over time they have accreted evermore peaks (childhood, student, labour, retirement, post-retirement [39]), which in turn can make them difficult to calibrate due to their large number of parameters (the most parsimonious has seven [47]). Furthermore, as the group interval increases, the user is confronted with the problem of controlling this set of parameters in the face of a decreasing number of observations.

In light of these constraints and issues, here we aim to develop a general framework for estimating migration probabilities from age-specific origin-destination matrices that can be used as the basis of a methods protocol for a Human Internal Migration Database (HIMD). Our approach is to generalise a recent method for smoothing single year of age data [56, 57] to the case of grouped ages. This new method combines the strengths of both splines and model migration schedules (flexibility, ease of calibration, ability to specify views on the reasonable form of the age distribution), accounts for sample noise, is stable when the number of age intervals becomes small and can be applied to a general age abridgement structure. We test this method to assess whether it is an improvement over an existing method both in terms of measures of goodness of fit to the observed data and plausibility of the generated profiles. From this, we propose a migration database methods protocol and illustrate its utility by generating complete schedules of internal migration cubes for 54 countries.

In the next section we introduce the problem of estimating probabilities from age-grouped data, its decomposition into the two problems of estimating generation and distribution components, and the solutions to these two problems using P-TOPALS and P-spline methods respectively. The data and methods section describes the preparation of migration data from IPUMS-I microdata samples, and the process of estimating complete migration schedules from grouped data using P-TOPALS/P-splines and a comparison method. In the results section we compare the two methods using metrics of best-fit and demographic plausibility. In the final two sections we discuss the implications of our results and conclude with possible directions for future development.

## Grouped migration probabilities

Assume a country has been divided into *d* + 1 geographical subunits and consider the internal movement of its population over a time interval *n* from a given origin *O* to the *d* possible destinations *D* which we index from 1 to *d*. Grouped migration data of transition type consists of counts

Mjnb=M1jnb⋮Mgjnb,j=1,…d, (1)

of Mijnb migrants to destination *j* in the age group [*a*_*i*_+*n*,*a*_*i*_+*n*+*b*_*i*_) out of an exposed population

Nb=N1b⋮N1b (2)

of _*b*_*N*_*i*_ in the age group [*a*_*i*_,*a*_*i*_+*b*_*i*_) for *i* = 1,…,*g*. The final age interval can be open (*b*_*g*_ = ∞) or closed (*b*_*g*_ < ∞). Age groups are usually contiguous (*a*_*i*+1_ = *a*_*i*_+*b*_*i*_) but the methods described here also apply when some or all of the intervals overlap or are disjoint. The population *b*^*N*^ is the number of people who are at origin *O* at the beginning of the time interval and who are also in the country at the end of the interval. It therefore excludes people who are not alive or who are not in the country after time *n*. Our aim is to use Mjnb and *_b_N* to estimate the probability mxjn, defined to be the probability a person initially aged *x* at origin *O* is in destination *j* after the time interval *n*, for all ages *x* = 0,1,…,*ω*. To be precise, mxjn are probabilities conditional on survival and remaining in the country, but we will refer to them simply as probabilities. We take the approach of Dyrting & Taylor [57] and use the generation-distribution framework [58] to factor the (*ω*+1)×1 vector _*n*_*m*^*j*^ of probabilities of migrating to destination *j* into the product

mjn=mn×cjn, (3)

where the out-migration probability _*n*_*m* is defined to be the (*ω*+1)×1 vector probability of out-migrating from origin *O* irrespective of destination and the migration ratio _*n*_*c*^*j*^ is defined to be the (*ω*+1)×1 vector probability of migrating to destination *j* conditional on out-migrating from origin *O*. Here, and in the following, all matrix operations and functions act elementwise (obtained by operating one element of the matrix at a time) unless stated otherwise. Sample out-migration probabilities are calculated by taking the ratio

m˜nb=MnbNb (4)

of the total number of migrants

Mnb=∑j=1dMjnb (5)

and the initial population. Sample migration ratios c˜jnb are calculated by taking the ratio

c˜jnb=MjnbMnb (6)

of migrants to destination *j* and the total number of migrants. In the generation-distribution framework, estimating mjn from migration data is equivalent to estimating out-migration probability and migration ratios from sample probabilities m˜nb and c˜jnb.

This problem presents a number of difficulties for any estimation method. First, grouping can significantly obscure the age-dependence of migration because it averages probabilities over the age interval. Secondly, the number of sample probabilities is usually less than the number of probabilities to estimate, *g* ≤ *ω* + 1, by a factor approximately equal to the average group interval. Lastly, when regarded as a measurement of actual probabilities, sample probabilities will have components of sample noise, notwithstanding that age grouping is sometimes used to mitigate the effect of small populations. The P-TOPALS approach is well suited to handling these problems because, like model migration schedules, it is open to views on the plausible age profile of migration probabilities, it can generate estimates from even a moderate number of observations because it uses low-dimensional penalties, and it explicitly accounts for and adapts to sample noise by using maximum likelihood fitting.

### Estimating out-migration

Both the number of migrants and the exposed population within each age group can be written in terms of numbers in each single-year age group. It follows [47] that a grouped probability can be written as a weighted average of single-year of age probabilities

Mnb=w⋅mn (7)

where *A*⋅*B* denotes matrix multiplication and the *g* × (*ω* + 1) weight matrix *w* has elements

$$w_{i,x}=\left\{\begin{array}{cc} N_{x}{/}_{b}N_{i} & a_{i}\leq x<a_{i}+b_{i},\;i=1,\ldots ,g.\\ 0 & \text{otherwise} \end{array}\right.$$ (8)

A person exposed to the risk of out-migrating who is initially aged *x* will span the age range [*x*,*x*+*n*) over the interval *n*. Therefore, the multi-year probability mxn can be expressed in terms of implied one-year probabilities *m*_*x*_ through the expression

mxn=1−∏x≤k<x+n1−mk. (9)

Eqs (7) and (9) illustrate that multi-year transition-style age-grouped migration probabilities are related to implied one-year probabilities by a combination of geometric and arithmetic averaging so that the probability minb is an average of *m*_*x*_ over the age range [*a*_*i*_,*a*_*i*_+*b*_*i*_+*n*).

In the P-TOPALS approach [56] we represent the (*ω*+1)×1 vector of probabilities *m* = [*m*_0_,…,*m*_*ω*_]’ relative to a standard migration curve $\hat{m}$

$$m=\hat{m}\times \exp(B\cdot \theta )$$ (10)

where $\hat{m}$ is an (*ω* + 1) × 1 vector, *B* is an (*ω*+1)×*l* matrix of B-spline functions arrayed columnwise, and *θ* is an *l*×1 vector of spline weights. The number of B-splines *l* is determined by the number of spline knots [59]. The relational form of Eq (10) makes it possible, via the standard $\hat{m}$, to express views on the age structure of out-migration that are otherwise obscured by the effect of age-grouping over a multi-year interval. The spline weights *θ* are found by maximising the function

L(θ)=N′b⋅m˜nblogMnb−Mnb−λ2θ′⋅Dk′⋅Dk⋅θ (11)

with the first term equal to the log-likelihood assuming sample noise has a Poisson distribution and the second term controlling the smoothness of the B-spline component by penalizing *k*-order differences in *θ*. Maximising *ℒ*(*θ*) leads to a system of nonlinear equations which can be solved by iterated linear regressions [56]. The derivation of this iteration is given in S1 Appendix.

The P-spline approach uses a finely spaced set of spline knots and controls the amount of smoothing through the choice of the penalty size λ. We see from Eq (11) that, for small values of *λ*, the maximum of *ℒ*(*θ*) will be determined by the first term. In this case the solution will be be approximately Mnb≈m˜nb when knot spacing is finer than age-grouping, showing that for small penalties P-TOPALS will approximate an interpolation of the sample probabilities. When the penalty is large, the second term in Eq (11) will dominate and in this case the B-spline component *B*⋅*θ* will approximate a (*k*-1)-order polynomial [60]. As the penalty ranges from small to large the P-TOPALS fit changes from a fit using *l* parameters to an approximate fit using *k* parameters. The penalty can be chosen automatically by optimising an information criterion, such as the Akaike information criterion [61] or Bayesian information criterion [62], that balances the improvement in fit against the increased number of effective parameters [56].

### Estimating migration ratios

The age-grouped migration ratio for destination *j* is related to grouped out-migration Mnb and destination-specific out-migration mjnb through the expression

mjnb=mnbcjnb. (12)

It is difficult to estimate ratios cjn because there is an additional constraint that the sum of ratios over all destinations combined must equal one. We generalise the approach used by Dyrting & Taylor [57] for ungrouped ratios and introduce grouped conditional ratios cjnb defined to be the the probability of migrating to destination *j* conditional on not migrating to destinations 1,…,*j*-1. It is related to cjnb by the expression

cjnb=a1nb,j=1sjnb×ajnb,j=2,…,d−1sdnb,j=d (13)

where sjnb is the product

sjnb=∏1≤k<j1−aknb. (14)

Single-year conditional ratios *a*^*j*^ are similarly defined in terms of *c*. The advantage of using conditional ratios is that they are only subject to the condition 0≤ajnb,aj≤1,, and are also related by a weighted average similar Eq (7),

ajnb=Tjnb⋅aj. (15)

Details of the derivation of the weight matrix Tjnb are given in S1 Appendix.

In the method given in Dyrting & Taylor [57] the (1+*ω*)×1 conditional migration ratio vector is expressed in terms of B-splines

$$\mathrm{logit}\left(a^{j}\right)=B\cdot \phi^{j}.$$ (16)

and the spline weights *φ*^*j*^ are determined by maximising the penalised likelihood function

Laj=K′nb⋅a˜jnblogajnb+1−a˜jnblog1−ajnb−λ2ϕj′⋅D′⋅D⋅ϕj, (17)

where

Kjnb=∑k=jdMknb. (18)

The first term in Eq (17) is the log-likelihood function assuming migration to destination *j* follows a binomial process (a person has or has not migrated to *j*), and the second term penalises first-order differences in the spline weights. Similar to out-migration, the above equation can solved for *ϕ*^*j*^ using iterated regressions and the penalty set manually or determined by optimising an appropriate information measure such as the Akaike information condition with corrections [57, 63].

## Materials and methods

IPUMS International [IPUMS-I, 64, 65] is a project which inventories, stores, harmonises and makes available census and survey microdata from around the world. There are 149 microdata samples in IPUMS-I with variables that allow the calculation of sample migration probabilities between first-level (sub-national) administrative units. From these samples we derived counts of movers Mnb and the exposed population _*b*_*N* by age group. Table 1 gives a summary of the samples by country, showing the range of years, maximum number of destinations (*d*_max_), migration intervals (*n*), number of samples (S), and the range of values for the maximum finite age group (*b*_max_) in each case. Of the 54 countries, 3 (Ireland, Israel, and United Kingdom) have grouped ages, accounting for 10 samples. Also shown in Table 1 is the range of Whipple’s Index of digital preference (*W*), a method for measuring age heaping to identify variability in the quality of age reporting across regions or countries, for each country [49, 66, 67], and the number of samples (G) that have grouped ages or would require grouped ages because their Whipple index was greater than or equal to 110. The latter is the threshold used by United Nations [68] to categorise data as being either "highly accurate" or "fairly accurate" data (*W* < 110) or "approximate", "rough", or "very rough" (*W* ≥ 110).

**Table 1:** IPUMS-I samples with transition-style migration data.

Country	Years	*d*_max_	*n*	S	*b*_max_	*W*	G
Argentina	[1970, 2001]	23	5	3	1	[102, 107]	0
Bolivia	[1976, 2012]	8	5	4	1	[110, 145]	4
Botswana	[1981, 2011]	20	1, 5	4	1	[99, 119]	1
Brazil	[1991, 2010]	24	5	3	1	[102, 104]	0
Cameroon	2005	6	5	1	1	173	1
Canada	[1981, 2001]	9	1, 5	3	1	99, 100	0
Chile	[1982, 2017]	42	5	4	1	[99, 103]	0
China	1990, 2000	28	5	2	1	100	0
Colombia	[1985, 2005]	21	5	3	1	[105, 138]	2
Costa Rica	[1973, 2011]	6	5	4	1	[103, 120]	1
Dominican Republic	1981, 2010	22	5	2	1	111, 114	2
Ecuador	[1990, 2010]	13	5	3	1	[103, 132]	2
Fiji	[1976, 2007]	3	5	4	1	[101, 110]	1
Ghana	2000	9	5	1	1	183	1
Greece	[1971, 2011]	53	1, 5	5	1	[101, 107]	0
Guatemala	[1973, 2002]	21	5	4	1	[126, 164]	4
Haiti	1982, 2003	3	5	2	1	161, 169	2
Honduras	[1974, 2001]	17	5	3	1	[104, 128]	2
Indonesia	[1976, 2010]	26	5	8	1	[114, 222]	8
Iraq	1997	14	10	1	1	104	0
Ireland	[1981, 2011]	5	1	7	1, 5	100	6
Israel^*^	1972, 1983	7	5	2	5, 7	——	2
Kenya	1979, 2009	7	1	2	1	145, 146	2
Laos	2005	17	10	1	1	138	1
Malaysia	1991, 2000	12	5	2	1	113, 114	2
Mauritius	[1990, 2011]	9	5	3	1	[101, 103]	0
Mexico	[1990, 2015]	31	5	6	1	[113, 125]	6
Mongolia	2000	20	5	1	1	98	0
Mozambique	1997, 2007	10	1, 5	2	1	[117, 126]	2
Nepal	2001, 2011	13	5	2	1	187, 203	2
Nicaragua	[1971, 2005]	11	5	3	1	[112, 188]	3
Pakistan	1973	3	8	1	1	335	1
Papua New Guinea	1990	18	1	1	1	139	1
Paraguay	[1972, 2002]	13	5	4	1	[105, 112]	2
Peru	2007	24	5	1	1	109	0
Philippines	2000, 2010	75	5, 10	2	1	108, 109	0
Poland	2002	15	1	1	1	100	0
Portugal	[1981, 2011]	21	1, 5, 7	4	1	100, 101	0
Russia	2010	80	1	1	1	103	0
Senegal	[1988, 2013]	7	1, 5, 10	3	1	[101, 185]	2
Sierra Leone	2004, 2015	13	5, 14	2	1	243, 249	2
South Africa	2001	3	5	1	1	97	0
South Sudan	2008	9	1	1	1	176	1
Spain	[1981, 2011]	18	1, 5, 10	4	1	[98, 102]	0
Sudan	2008	14	1	1	1	248	1
Tanzania	[1988, 2012]	22	1, 10	3	1	[153, 189]	3
Trinidad & Tobago	[1990, 2011]	3	1, 5, 10	3	1	106, 107	0
United Kingdom	1991, 2001	11	1	2	2, 15	98	2
United States	[1970, 2015]	50	1, 5	7	1	[100, 103]	0
Uruguay	[1975, 2006]	18	5	4	1	[101, 107]	0
Venezuela	2001	21	5	1	1	102	0
Vietnam	[1989, 2009]	37	5	3	1	[98, 100]	0
Zambia	[1990, 2010]	7	1	3	1	[121, 128]	3
Zimbabwe	2012	9	10	1	1	110	1
Total				149			76

Years, the range of years; *d*_max_, maximum number of destinations; *n*, migration intervals; S, number of samples; *b*_max_, maximum closed age group interval; *W*, the range of values of Whipple’s index of digital preference; G, the number of samples with grouped data or requiring grouping to mitigate digital preference and age overstatement. Based on Census data obtained from IPUMS International [64, 65].

^*^The Whipple index is not given for Israel because *W* is defined for ungrouped data in the age range 23 to 63 [49] and the samples for Israel have data that is grouped in this age interval.

The migration dataset was divided into two subsets: *D*_*s*_, the 73 samples with ungrouped data, and *D*_*e*_, consisting of the 76 samples where the data was grouped or required grouping. For samples in *D*_*e*_ with *W* ≥ 110, age was aggregated into five-year age groups. Digital preference can sometimes be stronger for ages equal to multiples of 10 compared to odd multiples of 5. This can lead to populations aggregated into five-year age groups displaying a marked "sawtooth" pattern of alternating high and low values which is quantified by a value for the sawtooth index *ST* [69] greater than 1. The samples that were grouped due to a high Whipple index were then examined for preferences in reporting age with end digit 0 over end digit 5. If a preference was evident visually or if *ST* was high (*ST* ≥ 1.11), ages over a given value (usually 40 years but for some samples lower) were aggregated into ten-year groups. Samples with an open age group were examined for age overstatement using an index *OS* based on Coale & Kisker [70] equal to the population of the open age group as a proportion of the population over 70 divided by the same ratio for a reference population. For the latter we used the United Nations [71] estimate for that country and year. For samples with *OS* ≥ 100, the open age interval was not included. Data were deposited in an OSF repository, available to the reader from the link provided in [72].

For each combination of country, year, and migration interval in *D*_*e*_, total first-level internal migration probabilities (the probability of changing the first-level administrative unit of residence regardless of origin) were estimated using P-TOPALS with a flat standard ($\hat{m}=1$), quadratic B-splines with nodes spaced 2.5 years apart from age 0 to age 180, and a linear penalty (*k* = 1) with the penalty size chosen by optimising the Bayesian information condition. For each first-level administrative unit, out-migration was then estimated using the same configuration with the country-specific total out-migration as the standard. Destination-specific migration ratios were then estimated using P-splines with quadratic B-splines with nodes spaced 2.5 years apart from age 0 to age 180, a linear penalty, with the penalty size chosen by optimising the Akaike information condition with corrections. In total we estimated 1,205 origin-specific and 20,220 origin-destination-specific schedules of migration probabilities at single-year intervals from age 0 to age 110, covering 80 migration cubes for 33 countries.

As a comparison method we used the hybrid approach of Rogers et al. [51], originally specific to five-year age groups, here generalised to the case of arbitrary age grouping. In this method, the *i*th sample probability m˜ijnb=MijnbNib with a closed age interval (*b*_*i*_ < ∞) is taken as the value of the estimated probability at the mid point of the age group

mxijn=m˜ijnb,xi=ai+bi2. (19)

Probabilities at single-year ages between the first and last mid point are found using the *x*_*i*_ as knots of a cubic spline. Final estimates at all ages are then found by fitting the spline with the student-peak model migration schedule [54]

mxjn=a1exp−α1x(childhood)+a2exp−α2x−μ2−e−λ2x−μ2(labourforce)+a3exp−x−μ3σ32(retirement)+a4expα4x(elderly)+a5exp−α5x−μ5−e−λ5x−μ5(student)+c(constant). (20)

Origin-destination-age-specific migration probabilities were estimated by the hybrid method using the following. Step 1: best-fit parameters were obtained for total first-level migration. Step 2: parameters from Step 1 were then used as starting values for an estimation of the best-fit parameters for origin-age-specific out-migration. Step 3: parameters from Step 2 were used to estimate best-fit parameters for origin-destination-age-specific migration by adjusting the level parameters (*a*_1_,*a*_2_,*a*_3_,*a*_4_,*a*_5_, and *c* in Eq (20)), keeping the other parameters fixed at the origin-specific values. If any non-constant component in Eq (20) was not used in a step (for example, by holding its level *a*_*i*_ fixed at 0) then it was not used in the subsequent steps.

**Fig 1:**
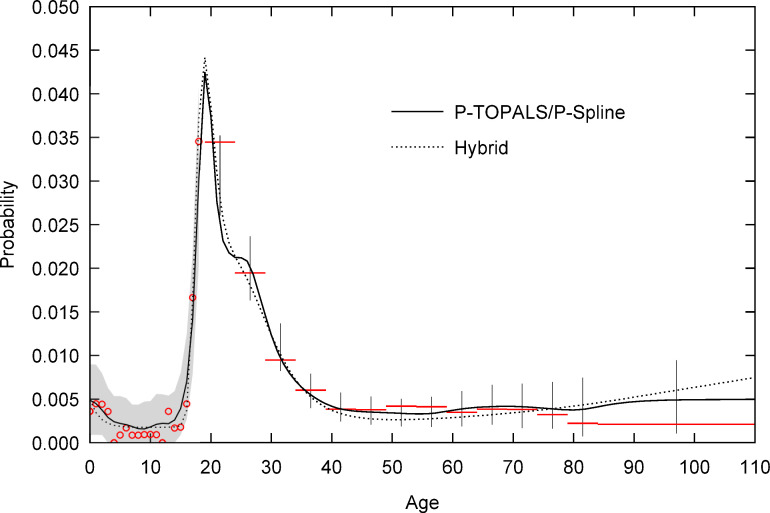
Age specific 1-year migration probability from the Mid-West and South-East to Dublin by estimation method, Ireland 2006. Sample probabilities are single-year of age (circles) and grouped (horizontal lines). Grey area and vertical lines indicate 95% confidence interval for sample probabilities based on P-TOPALS/P-spline fit. Age is measured at the start of the migration interval. The open age group begins at age 84. Source: Author calculations based on Census data obtained from IPUMS International [64, 65].

**Fig 2:**
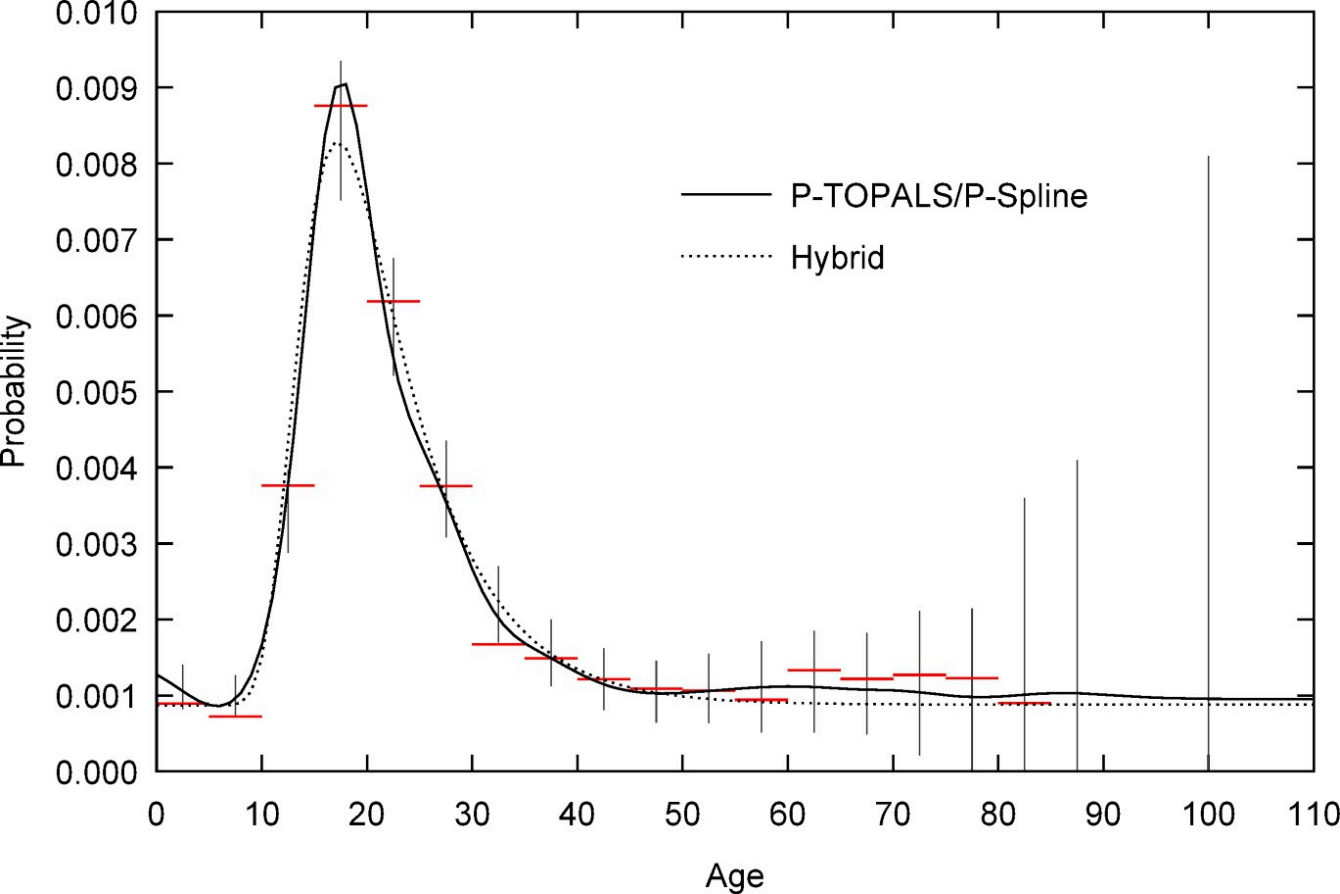
Age specific five-year migration probability from Sumatera Barat to DKI Jakarta by estimation method, Indonesia 2010. Horizontal lines, grouped sample probabilities; vertical lines, 95% confidence interval for sample probabilities based on P-TOPALS/P-spline fit. Age is measured at the start of the migration interval. The open age group begins at age 90. Sample probabilities for the 85–89 and 90+ age groups are zero. Source: Author calculations based on Census data obtained from IPUMS International [64, 65].

Figs 1 and 2 respectively illustrate the two cases where migration probabilities are estimated by expanding data that is published in grouped form (Ireland, 2006) or requires grouping because of age heaping (Indonesia, 2010). Evident in both figures are the significant amounts of sample noise both in the probabilities at single year of age (Fig 1, ages 0 to 15) and groups (closed and open).

## Results

We compared the two methods using three metrics: two measures of the level of fitting error between sample and estimated probabilities, and a measure of deviation of the estimated probabilities from a reference schedule. The first goodness-of-fit statistic, which we call the migration fitting error, is the scaled multinomial deviance measuring the aggregate error of estimates of all migration probabilities from a given origin

dev=2gd×N′b⋅1−m˜nblog1−m˜nb1−mnb+∑j=1dm˜jnblogm˜jnbmjnb. (21)

**Fig 3:**
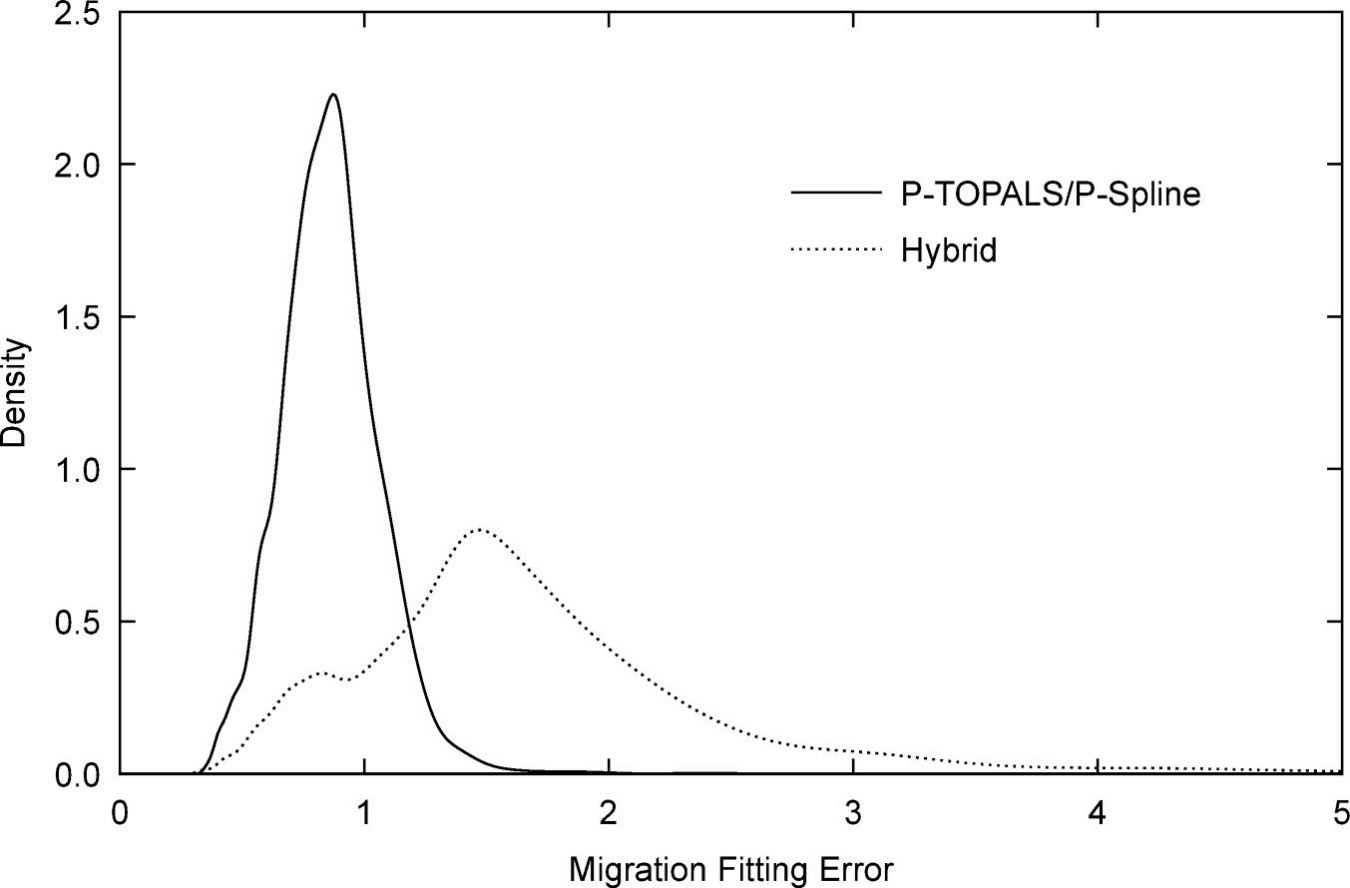
Migration fitting error density by estimation method. Migration fitting error (dev) is given by Eq (21). Source: Author calculations based on Census data obtained from IPUMS International [64, 65].

In this expression the scaling factor 1/(*gd*) has been added to control for the effect of the number of observations and destinations on the total deviance and allow comparisons between cases with different age groupings and spatial decompositions. Deviance can be thought of as the generalisation of the sum of squared standardised errors to case of a general distribution, in this case the multinomial distribution. Fig 3 shows the migration fitting error densities for P-TOPALS/P-spline and the hybrid method calculated using kernel density estimation [73]. We see that, compared to the hybrid method, P-TOPALS/P-spline fitting errors have a much lower mean (0.86 compared to 1.97 for the hybrid method) and smaller standard deviation (0.20 compared to 2.86 for the hybrid method). These results indicate that the P-TOPALS/P-spline approach provides a consistently better fit to destination-specific probabilities.

For a given origin, a good fit of migration probabilities to all destinations does not necessarily lead to a good fit of total out-migration from a given origin. To assess this, we use an additional goodness-of-fit measure, the scaled binomial deviance for total out-migration

devm=2g×N′b⋅1−m˜nblog1−m˜nb1−mnb+m˜nblogm˜nbmnb. (22)

**Fig 4:**
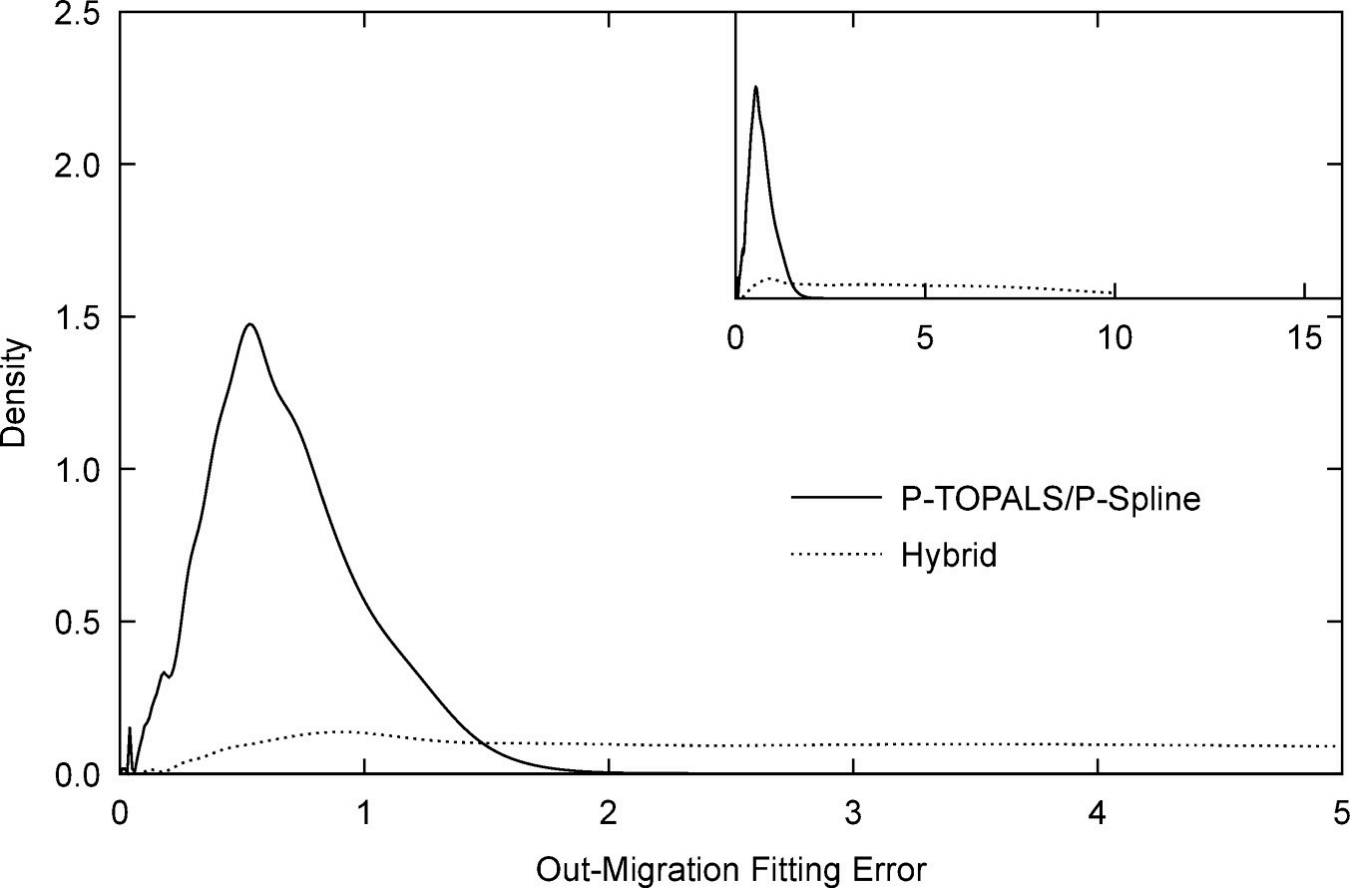
Out-migration fitting error density by estimation method. Out-migration fitting error (dev_*m*_) is given by Eq (22). Note: Inset uses an expanded scale to show the broad distribution of fitting error for the hybrid method. Source: Author calculations based on Census data obtained from IPUMS International [64, 65].

Fig 4 shows the out-migration fitting error densities for P-TOPALS/P-spline and the hybrid methods. We see that, compared to the hybrid method, P-TOPALS/P-spline out-migration fitting errors also have a lower mean (0.69 compared to 9.30 for the hybrid method) and smaller standard deviation (0.31 compared to 34.22 for the hybrid method).

For cases where the number of observations *g* is of the same order as the number of fitting parameters, it is possible for a method to achieve an accurate fit to sample probabilities with an unrealistic profile. To assess the plausibility of the estimated destination-specific migration probabilities we use the shape deviation metric $\bar{P}$, equal to the sum of the percentage that each destination-specific schedule’s profile differs from a reference profile (_*n*_*m*_ref_)

P¯=∑j=1d1001−mref′n⋅mjn|mrefn||mjn. (23)

**Fig 5:**
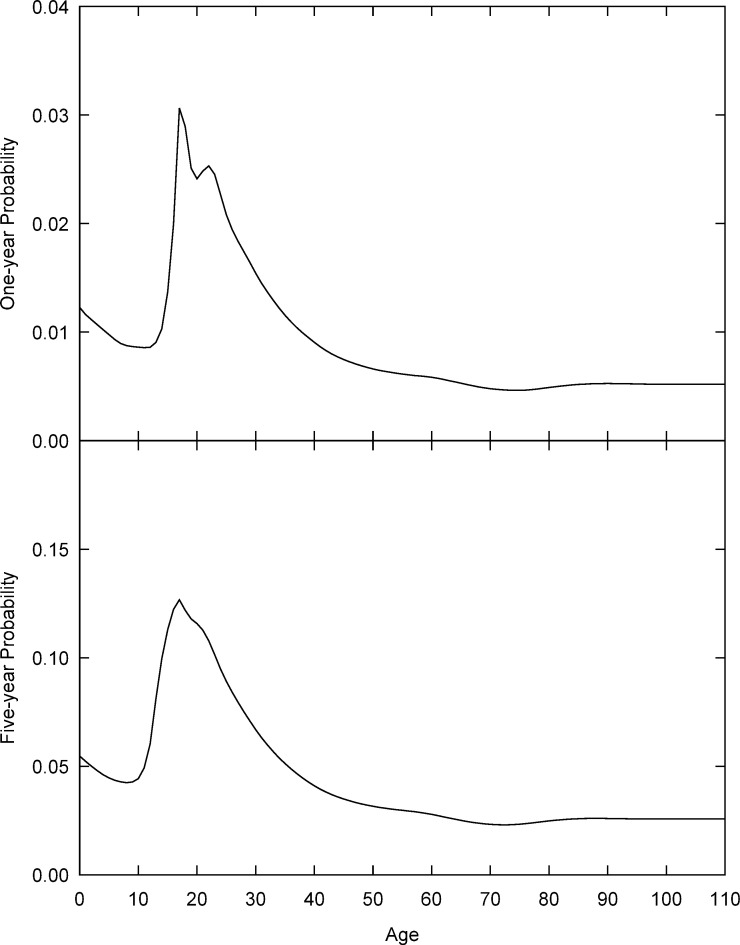
One-year and five-year reference migration schedules. Source: Author calculations based on Census data obtained from IPUMS International [64, 65].

The reference profiles _*n*_*m*_ref_ were calculated from the ungrouped dataset *D*_*s*_ as follows. For each of the 2235 combinations of first-level administrative regions and migration intervals, we used the method given in Dyrting [56] to estimate a smooth schedule of implied one-year out-migration probabilities, rescaled to sum to 1 over ages 0 to 110. The one-year reference schedule *m*_ref_ was defined as the average of the scaled implied one-year curves, and multi-year reference schedules _*n*_*m*_ref_ calculated from *m*_ref_ using Eq (9). Fig 5 shows the resulting reference curves for the two most frequent intervals, *n* = 1 and *n* = 5. We see that both the one-year and the five-year probabilities have a prominent labour peak centred near age 20. The one-year probabilities also display a student peak at age 17, which is also evident in the five-year probabilities as a discontinuous change in the slope [56].

**Fig 6:**
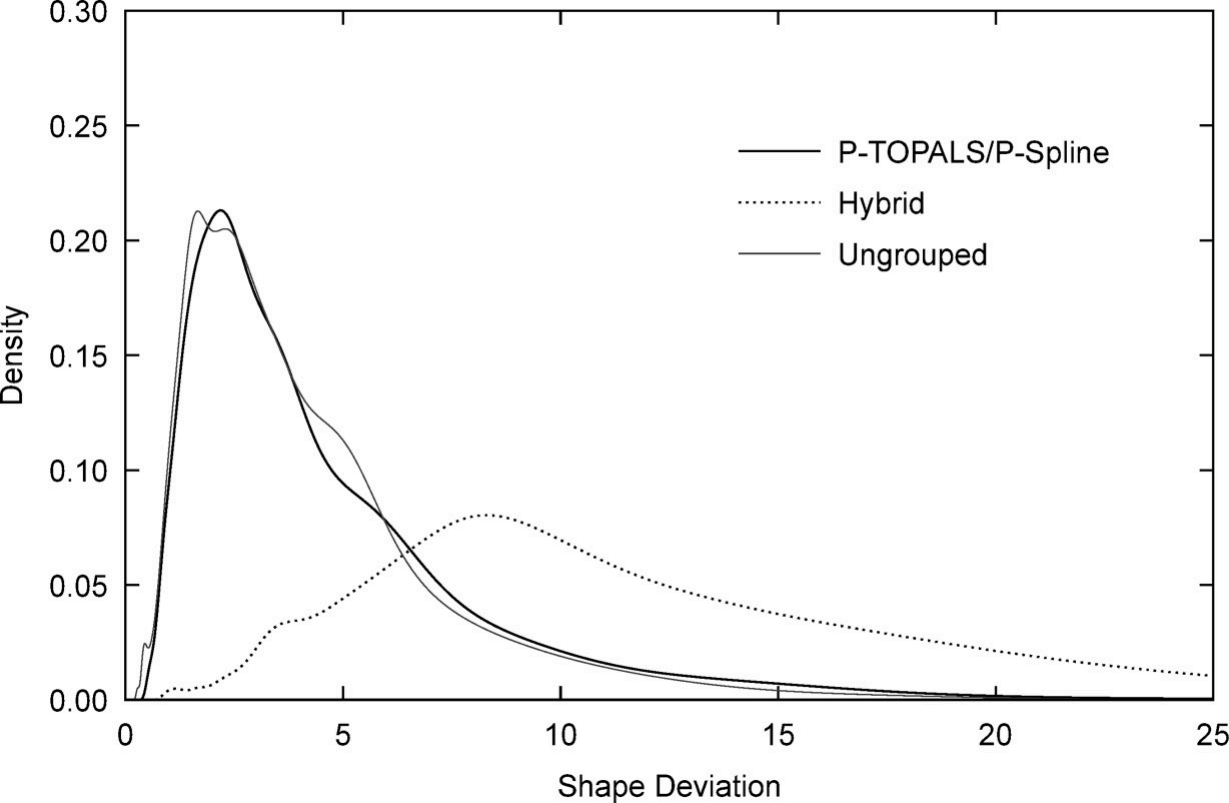
Shape deviation by estimation method. Shape deviation ($\bar{P}$) is given by Eq (23). Source: Author calculations based on Census data obtained from IPUMS International [64, 65].

Fig 6 shows the shape deviation densities for P-TOPALS/P-spline and the hybrid methods, and, for reference, the density of the shape deviation for the ungrouped dataset *D*_*s*_. It shows that P-TOPALS/P-spline deviations are concentrated at lower values (mean 4.81 and standard deviation 3.92) compared to the hybrid method (mean 13.38 and standard deviation 8.63) and have a distribution similar to the ungrouped dataset (mean 4.35 and standard deviation 3.13), showing that the variation in expanded schedules generated by P-TOPALS/P-splines is plausible, and consistent with the range of shapes present in smoothed ungrouped data.

To further test the relative performance of P-TOPALS/P-spline and the hybrid method we compared expansions of Australian census 2021 interstate migration data grouped over 5 and 10 year age intervals with data at 1 year age intervals, all of which are available from the Australian Bureau of Statistics [74]. Table 2 gives migration and out-migration goodness of fit measures by state, migration interval, and age grouping. It shows that P-TOPALS/P-spline has the smallest migration fitting error in 30 cases and the hybrid method has the smallest error in 2 cases (ACT and South Australia, 1-year migration interval and 10-year age grouping). P-TOPALS/P-spline has the smallest out-migration fitting error in 29 cases and the hybrid method has the smallest error in 3 cases (New South Wales, 1-year migration interval, 5-year and 10-year age groups, and Tasmania, 1-year migration interval, 10-year age group).

**Table 2:** Summary statistics for Australia 2021 case study.

State	*n*	*b*	dev^*P*^	dev^*H*^	${\mathrm{dev}}_{m}^{P}$	${\mathrm{dev}}_{m}^{H}$
Australian Capital Territory	1	5	1.97	2.03	3.28	3.82
	1	10	2.35	2.18	5.65	6.78
	5	5	1.56	8.71	1.61	15.15
	5	10	1.82	8.85	2.51	16.82
New South Wales	1	5	3.97	8.32	16.44	15.04
	1	10	5.70	9.23	22.33	22.16
	5	5	2.70	22.51	7.70	33.61
	5	10	5.12	24.68	12.86	36.15
Northern Territory	1	5	1.46	2.68	2.45	3.40
	1	10	1.66	2.38	3.70	3.70
	5	5	1.55	6.82	1.90	8.77
	5	10	1.76	6.63	2.02	8.96
Queensland	1	5	2.14	3.27	6.27	6.61
	1	10	2.65	4.94	8.00	10.35
	5	5	1.69	9.98	3.17	21.36
	5	10	2.11	13.36	3.61	30.30
South Australia	1	5	1.86	1.88	3.70	3.97
	1	10	2.05	1.97	4.67	5.14
	5	5	1.56	4.36	2.38	5.88
	5	10	1.90	4.37	4.37	7.01
Tasmania	1	5	2.09	2.28	3.76	4.56
	1	10	2.69	2.81	7.41	7.22
	5	5	1.62	5.06	1.87	5.16
	5	10	2.07	4.59	4.94	7.29
Victoria	1	5	1.96	4.12	5.11	7.77
	1	10	2.46	7.81	6.43	15.45
	5	5	1.79	18.98	1.85	22.87
	5	10	3.26	19.34	7.64	54.04
Western Australia	1	5	1.87	2.46	3.58	4.27
	1	10	2.23	3.35	5.00	7.97
	5	5	1.54	6.55	2.14	15.11
	5	10	1.77	6.83	2.61	18.97

State, the state of origin; *n*, migration interval; *b*, closed age group interval; *dev*^*P*^, migration fitting error for PTOPALS/P-spline expansion; dev^*H*^, migration fitting error for the hybrid method; ${\mathrm{dev}}_{m}^{P}$, out-migration fitting error for PTOPALS/P-spline expansion; ${\mathrm{dev}}_{m}^{H}$, out-migration fitting error for the hybrid method. Migration and out-migration fitting errors are given by Eqs (21) and (22) respectively, with *b* = 1. Based on Census data obtained from the Australian Bureau of Statistics [74].

**Fig 7:**
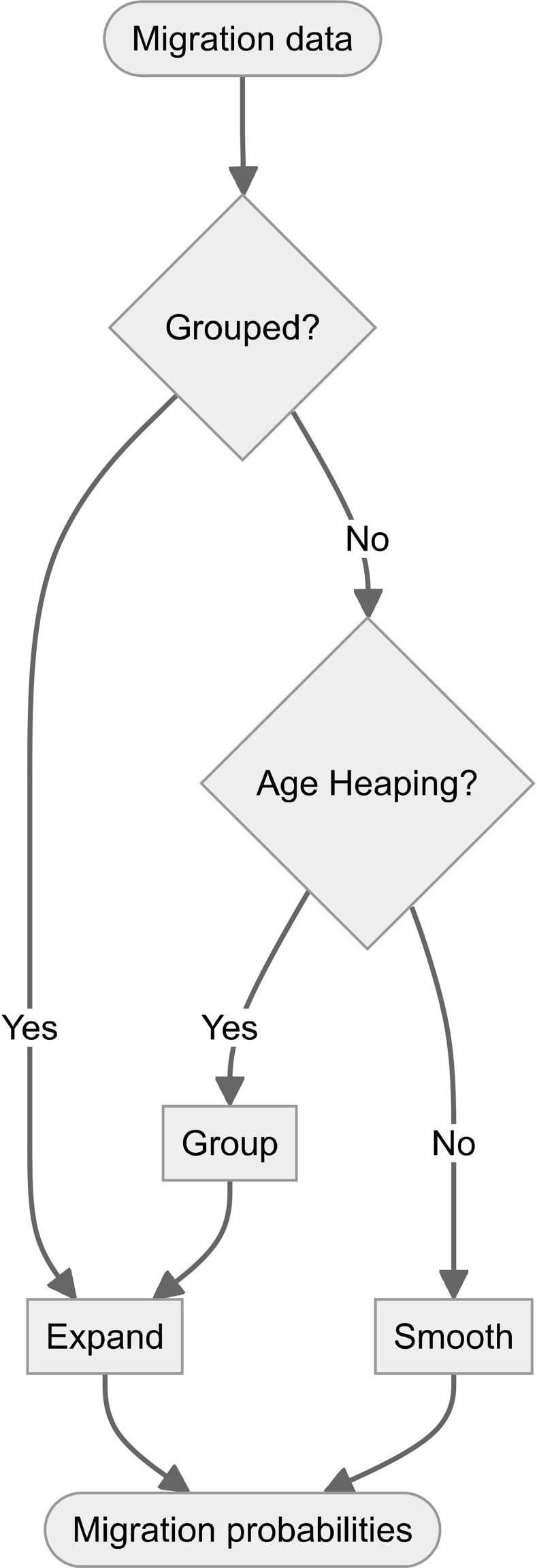
Proposed HIMD protocol flow diagram.

We now turn to describing our HIMD methods protocol which is an application from the above. A HIMD methods protocol for estimating complete schedules from transition-style data will need to include processes for smoothing single-year data and expanding grouped data. Fig 7 gives a summary of our proposed protocol using the P-TOPALS/P-spline method and applied to migration data *D* to illustrate a proof-of-principle. We used the method given in Dyrting & Taylor [57] to smooth migration ratios for each origin in *D*_*s*_ and combined the results with the smoothed out-migration estimates described above to produce 3,468 origin-specific and 73,067 origin-destination-specific schedules of migration probabilities at single-year intervals from age 0 to age 110. These schedules were combined with the expanded schedules from *D*_*e*_ to give a set of 172 complete migration cubes for 54 countries. These internal migration probabilities are available for download from an Open Science Foundation repository [72].

## Discussion

We have shown that the methodology for estimating migration probabilities to develop a HIMD will need to account for both ungrouped and grouped migration data. We find that of the 149 census samples available in IPUMS-I, approximately half (76) used grouped ages or required grouping to mitigate age misstatement. We have extended the P-TOPALS/P-spline smoothing method to the problem of expanding grouped data and shown that, as an expansion method, it is both more accurate and better able to produce plausible schedules than an existing approach based on cubic splines and model migration schedules. We illustrated the feasibility of the method by creating complete first-level migration cubes from sample data in IPUMS-I covering 54 countries.

As well as being a useful method for both smoothing and expanding migration data, the P-TOPALS/P-spline method is also a useful tool for harmonising migration data for different transition intervals to a common representation, 1-year implied out-migration probabilities (Eq (9)) and conditional ratios (Eq (15)). We made use of this in the Results section to derive reference schedules. Additional possibilities for expanding this work include extending the calibrated splines (CS) methodology [75, 76] to migration by performing shape-calibration using the HIMD 1-year implied out-migration probabilities, and investigating how implied out-migration changes with interval length for the countries that record changes of address over more than one interval (Botswana, Canada, Greece, Mozambique, Philippines, Senegal, Spain, Trinidad and Tobago) to gain further understanding of the 1-year/5-year problem [36, 37, 77].

**Fig 8:**
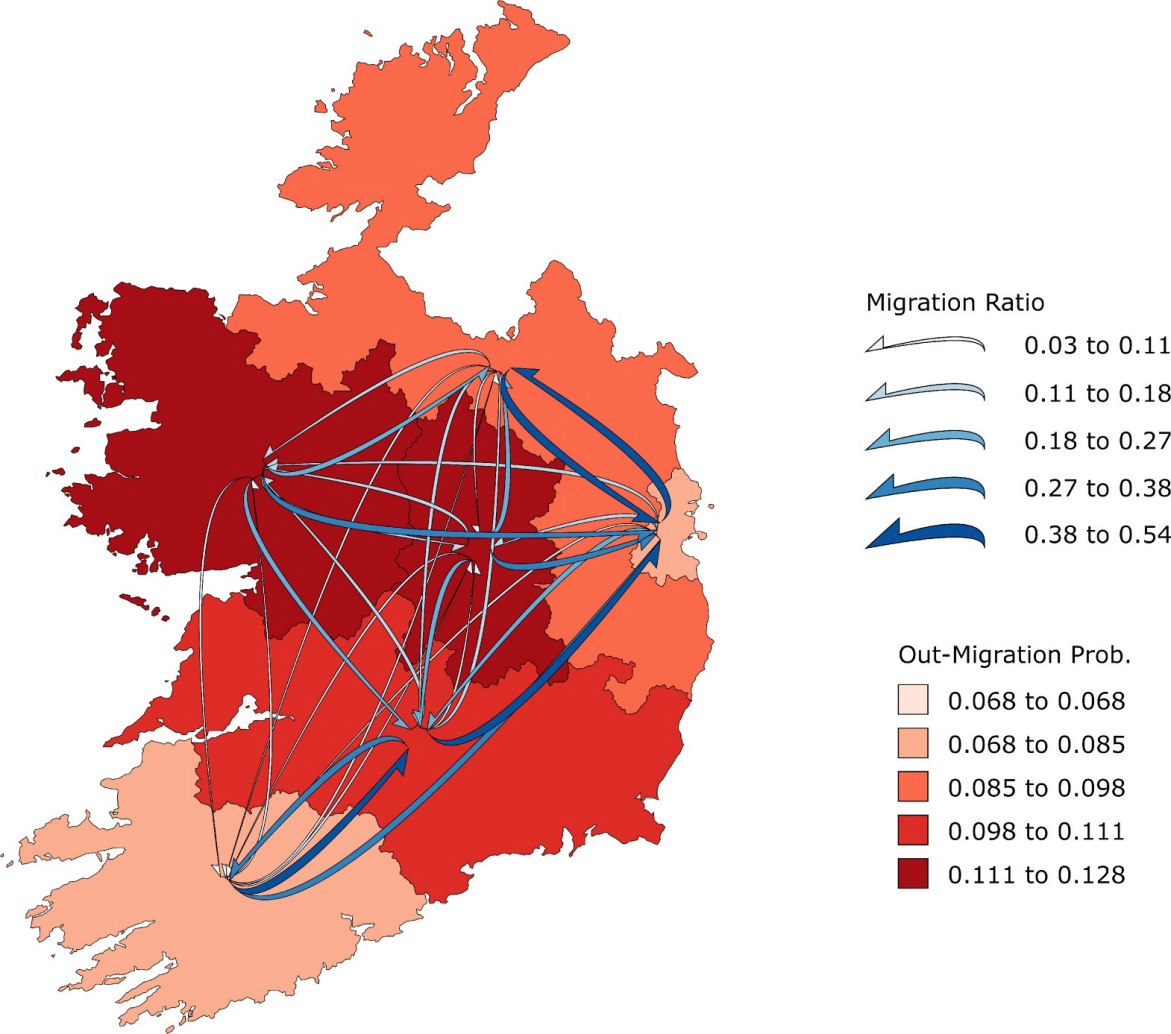
One-year internal migration probabilities at age 20, Ireland 2006. Spatial decomposition of Ireland is by IPUMS International harmonised first-level geography. The shapefile can be accessed at https://international.ipums.org. Source: Author calculations based on Census data obtained from IPUMS International [64, 65].

**Fig 9:**
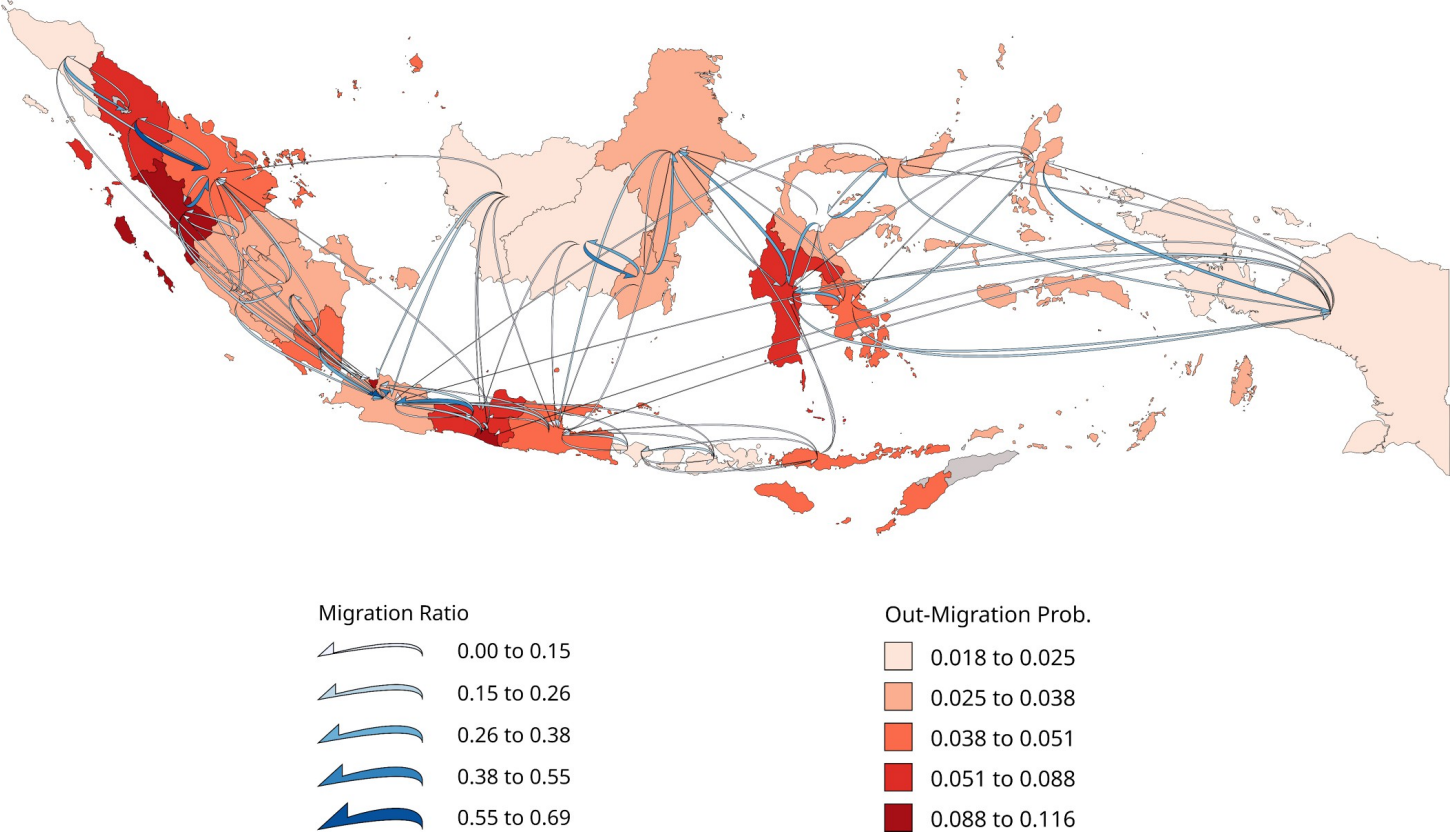
Five-year internal migration probabilities at age 20, Indonesia 2010. Spatial decomposition of Indonesia is by IPUMS International harmonised first-level geography. Only the top 100 migration ratios are shown. The shapefile can be accessed at https://international.ipums.org. Source: Author calculations based on Census data obtained from IPUMS International [64, 65].

An effective protocol for producing migration cubes from census data also has positive implications for the use and dissemination of migration statistics. For example, it will make it easier to specify the migration matrix input to multiregional population projection models, considered as the benchmark for intra-national and national population projections [78]. In addition, national statistics offices regularly publish life tables, and our methodology could be used to produce complete migration probability cubes for them to provide as one element of their census data release program. Furthermore, a challenge for spatial demography is the graphical summary of both age and spatial structure. Figs 8 and 9 reprise Figs 1 and 2 with flow maps produced using FlowMapper.org [79] and highlight the spatial pattern of out-migration and inter-region connectivity but not the age structure. It is our hope that a HIMD will encourage interactions between demographers and cartographers to develop automated processes for representing both the spatial and age patterns of migration [79–81]. The work here provides a stepping-off point for this to occur by providing a harmonised common data source.

Our finding that P-TOPALS/P-spline out-performs the hybrid method in both accuracy and plausibility is consistent with the strengths of the former method and the limitations of the latter. While P-TOPALS/P-spline treats grouped probabilities exactly (Eq (7)), the hybrid method approximates them by the migration probabilities in the centre of the age group and does not account for the open interval (Eq (19)). P-TOPALS/P-spline takes a top-down approach which ensures that estimates of destination-specific migration are consistent with estimates of out-migration, whereas the hybrid method is bottom-up, estimating destination-specific probabilities independently, so that a small value for dev does not imply a small value for dev_*m*_. As the exposed population decreases, sample noise increases. In this case, P-TOPALS/P-spline responds by decreasing the effective number of fitting parameters. In contrast, the number of parameters in the hybrid model is fixed, and the parameter values become less constrained as sample noise increases. In some cases this can lead to the generation of implausible schedules using the hybrid method.

Despite the better performance of P-TOPALS/P-spline, we acknowledge that the method has drawbacks which might cause researchers to prefer the hybrid method. The algorithms given in the subsections on estimating out-migration and migration ratios are more complex than the process for fitting the hybrid method given in the Methods and Materials section, and we found implementing P-TOPALS/P-spline in software required more work than adapting model migration schedules for grouped data. In our case the method supports a multi-country database which we feel justifies the additional work, but researchers undertaking smaller studies might feel it does not. Once the method was implemented it was then necessary to decide on the configuration of parameters to apply such as the choice of standard, the fineness of the the knot spacing, the degree of the B-splines, the order of the penalty, and the method for choosing the optimal penalty size. While, for the hybrid method, it is also necessary to decide which parameters to fix and which to fit, users will be more familiar with this choice given model migration schedules have been used for nearly 50 years [6].

The P-TOPALS/P-spline configuration outlined in the Methods and Materials section was applied to all samples in *D*_*e*_. There is, therefore, the potential for improvements to be made by designing configurations that are specific to the sample. For example, the choice of a flat standard ($\hat{m}=1$) when estimating the total first-level migration probability will imply a smoothly varying profile across ages where the group interval *b*_*i*_ is large. If there are reasons to believe migration probabilities are not smooth across an age group, for example it includes a student peak, then this can be incorporated by choosing a standard that includes this feature. The size of the penalty that controls the amount of smoothing was determined by the Bayesian information criterion for out-migration and by the Akaike information criterion for migration ratios. If there are reasons to believe sample noise is being under- or over-smoothed, then an alternate criterion can be used, or the the penalty set manually. The weights *w*_*i*,*x*_ in Eq (8) depend on the exposure *N*_*x*_ at age *x* which was estimated by a spline interpolation of the grouped exposures _*b*_*N*_*i*_ that assumes an exponentially decreasing profile over open intervals. It may be that the user has additional information on the age distribution which can be used in the calculation of *N*_*x*_ by expanding _*b*_*N*_*i*_ using P-TOPALS for age distributions [82].

Progress has been slow in the development of methods for expanding grouped migration data beyond the spline and MMS approaches first introduced in Rogers et al. [47]. Up to now these two methods have remained the standard, though now combined into a hybrid approach [51]. Consequently, there has been a dearth of comparative studies for alternate methods and countries. This is likely a result of the estimation of migration probabilities requiring the careful handling of multiple factors that, in combination, make the problem formidable. First, the number of schedules to estimate is quadratic in the number of spatial units (*d*), becoming large for even first-level administrative units, and making manual intervention unfeasible and small-sample noise unavoidable. Secondly, small sample noise cannot be reduced by pooling consecutive years because data is collected infrequently. Thirdly, data is often doubly abridged, spanning multiple years of both time (*n* > 1) and age (*b* > 1). Our findings show that it is possible to address these challenges within the P-TOPALS/P-spline framework, adding non-parametric methods to the demographer’s migration toolbox, and illustrating the feasibility of a HIMD dataset which will be a useful testing ground for further advances in estimation methods and will enhance demographers’ and other experts’ access to quality data for modelling human spatial exchanges.

In this article, the population exposed to internal migration is conditional on being in the country at the start and at the end of the time interval. It therefore excludes people who are international migrants (people who are outside the country at either the start or end of the interval) which reduces the potential for direct biases in estimates of internal migration probabilities due to regional variations in international migration rates. This is not to say that international migration does not affect internal migration. International migrants (people born outside the country) will be included in internal migration rates provided they are in the country at the start and at the end of the interval, and to the extent that they tend to prefer large urban centres, they will influence urban-regional migration probabilities [83, 84].

There are a number of limitations of the migration dataset we used in this study that have implications for the utility of our estimated migration cubes. IPUMS-I datasets are census microdata samples and as such will include higher levels of sample noise than the full census microdata. Access to the complete census data would allow more accurate estimates of migration probabilities but would also entail greater costs in time and access fees. Table 1 shows that our dataset includes most countries in the Americas but under-represents Africa, Europe, Asia, and Oceania. Further work needs to be done collecting data for these regions to make the dataset reflect a global view of internal migration. Of the 54 countries, 49 have less than 5 census years represented in our dataset, and none are prior to the 1970s. This constrains the level of time series analysis that can be performed. Comparisons between countries are complicated by differences in migration interval *n* mentioned in the Introduction. Currently our dataset estimates probabilities of migrating between first-level administrative regions. Analysis of migration between second-level regions, or between urban and regional areas, will require data at finer spatial levels.

A global Human Internal Migration Database will provide information on the movement of subnational populations and can therefore have significant ethical and practical implications. Publishing data on small populations raises concerns that information about an individual might be disclosed even though the data is aggregated [85]. PTOPALS/P-spline is a mapping from aggregate counts of movers and exposed population to probabilities that are smoothed across ages. The amount of smoothing we have applied increases as the exposed population decreases. As a result, the method here is comparable to the types of data modification techniques applied to census and other data by national statistical agencies to prevent identification and disclosure of individuals or groups of individuals who may be vulnerable [40, 86].

## Conclusions

We have shown that it is possible to extend both P-TOPALS and P-spline methods for smoothing migration probabilities and ratios [56, 57] to the problem of expanding grouped migration data. We find that it out-performs the hybrid spline-MMS method both in terms of fitting sample data and plausibility of the expanded schedules. Furthermore, we have shown that it is feasible to construct a database of complete schedules of internal migration cubes using the same framework for both smoothing data of high quality and expanding data that has been grouped to mitigate age misstatement that can form the core of a HIMD. These migration cubes are available for download from an Open Science Foundation repository [72].

A limitation of the method developed in this article is that it can only be applied to transition-style data collected from population censuses or surveys. A significant number of countries collect event-style migration data using population registers [38]. Consequently, a direction for further research is to extend the methods here to estimate migration schedules from event-based sample rates.

We have applied the P-TOPALS/P-spline method to estimating migration probabilities between first-level administrative units, but the methods can also be applied at more granular geographies. For example, IPUMS-I provides migration data for second-level geographic subunits for a selection of countries. The strategy we used in the Results section of first estimating aggregate migration, and then using it as a standard for estimating origin-specific out-migration can also be applied in this case. Sample noise would increase relative to the first-level case as a result of the reduction in exposed populations, and the penalty terms in Eqs (11) and (17) will play an increasing role in the expansion of probabilities and ratios.

Although in this study our method was shown to perform well against the hybrid method, we believe that model migration schedules will continue to be a valuable tool for studying migration. However, it will be necessary to solve the problem of the automatic solution of optimal fitting parameters for them to be useful for analysing large datasets. It has long been recognised that the MMS parameters can be grouped into families [47, 51, 87], and it might be possible to use the range of profiles spanned by the schedules estimated in this article to specify additional constraints on the parameters that regularise the MMS fitting problem in a way analogous to the shape constraints used in mortality calibrated splines [76].

## Supporting information

S1 Appendix Solving for the B-spline weights. (DOCX)
