# Supplementary material for: Estimating complete migration probabilities from grouped data: A methods protocol for developing a global Human Internal Migration Database
Source: PLoS One. 2024 Dec 10;19(12):e0315389. doi: 10.1371/journal.pone.0315389 (PMC11630604; doi:10.1371/journal.pone.0315389)
Supplement: S1 Appendix — (DOCX) [file pone.0315389.s001.docx]

# S1 Solving for the B-spline weights

## Solving the Out-Migration Equations

Following [[1](#ref-dyrting2020)], the spline weights $\theta$ that maximise the log-likelihood function defined by Eq (11) satisfy a nonlinear system of equations obtained by setting the derivative of $\mathcal{L}\left( \theta\right)$ to zero. Using a linear approximation to the change in ${}_{n}^{b}m$ as a function of a change in spline weights $\theta-\theta$ and substituting into the nonlinear equations gives the linear equation

$$Q\left( \theta\right)\cdot\theta=r\left( \theta\right) \left( S1-1 \right)$$

for the updated weights $\theta$ given the current approximation $\theta$, where

$$Q\left( \theta\right)=F'\left( \theta\right)\cdot W\left( \theta\right)\cdot F\left( \theta\right)+\lambda D_{k}'\cdot D_{k} \left( S1-2 \right)$$

$$r\left( \theta\right)=F'\left( \theta\right)\cdot V\cdot\left( {}_{n}^{b}\tilde{m}-{}_{n}^{b}m \right)+F'\left( \theta\right)\cdot W\left( \theta\right)\cdot F\left( \theta\right)\cdot\theta\left( S1-3 \right)$$

and

$$W\left( \theta\right)=diag\left( {}_{n}^{b}m\times{}_{b}N \right), \left( S1-4 \right)$$

$$V=diag\left( {}_{b}N \right). \left( S1-5 \right)$$

and $F$ is given by

$$F=w\cdot G \left( S1-6 \right)$$

where $w$ is the weight matrix with elements given by Eq (8) and

$$G_{x}\left( \theta\right)=\frac{1-{{}_{n}m}_{x}}{{{}_{n}m}_{x}}\left( \sum_{x\leq j<x+n} \frac{m_{j}}{1-m_{j}}B_{j} \right). \left( S1-7 \right)$$

## Solving the Migration Ratio Equations

Since both ${}_{n}^{b}m^{j}$ and ${}_{n}^{b}m$ are related to single-year probabilities by the weighted average Eq (7), it follows from Eq (3) and Eq (12) that ${}_{n}^{b}c^{j}$ can be written as a linear combination

$${}_{n}^{b}c^{j}=v\cdot{}_{n}c^{j} \left( S1-8 \right)$$

of single-year of age migration ratios ${}_{n}c^{j}$ where the $g\times\left( 1+\omega\right)$ weight matrix $v$ has elements

$$v_{i,x}=\left\{ \begin{matrix} \left( {{}_{n}m}_{x}\times N_{x} \right)/\left( {{}_{n}^{b}m}_{i}\times{{}_{b}N}_{i} \right) & a_{i}\leq x<a_{i}+b_{i} \\ 0 & \mathrm{otherwise} \end{matrix} \right.. \left( S1-9 \right)$$

If destination-specific migration is modelled as a multinomial process [[2](#ref-dyrting2021)] and out-migration rates have already been estimated, then the loglikelihood of the sample migration ratios is

$$\mathcal{L}_{c}={}_{n}^{b}M'\cdot\sum_{j=1}^{d} {}_{n}^{b}\tilde{c}^{j}\log\left( {}_{n}^{b}c^{j} \right). \left( S1-10 \right)$$

Finding the ${}_{n}c^{j}$ that maximise the likelihood Eq ([S1‑10](#eq:rloglik)) is difficult because there is an auxiliary condition that the ratios must sum to one,

$$\sum_{j=1}^{d} {}_{n}^{b}c_{i}^{j}=1. \left( S1-11 \right)$$

Substituting the expression Eq (13) for the ratios in terms of conditional ratios into Eq ([S1‑10](#eq:rloglik)) gives

$$\mathcal{L}_{a}=\sum_{j=1}^{d-1} \mathcal{L}_{a}^{j}, \left( S1-12 \right)$$

where

$$\mathcal{L}_{a}^{j}=\left( {}_{n}^{b}K^{j} \right)'\cdot y_{j}, \left( S1-13 \right)$$

$${}_{n}^{b}K^{j}=\sum_{k=j}^{d} {}_{n}^{b}M^{k}, \left( S1-14 \right)$$

and

$$y_{j}={}_{n}^{b}\tilde{a}^{j} \log{}_{n}^{b}a^{j}+\left( 1-{}_{n}^{b}\tilde{a}^{j} \right) \log\left( 1-{}_{n}^{b}a^{j} \right). \left( S1-15 \right)$$

Age-grouped multi-year conditional ratios ${}_{n}^{b}a^{j}$ can be expressed in terms of implied single-year ratios $a^{j}$ as follows

$${}_{n}^{b}a^{j}={}_{n}^{b}T^{j}\cdot a^{j}, \left( S1-16 \right)$$

where

$$\begin{matrix} {}_{n}^{b}T^{1} & =v\cdot{}_{n}U, \\ {}_{n}^{b}T^{j} & =diag\left( \frac{1}{1-{}_{n}^{b}a^{j-1}} \right)\cdot{}_{n}^{b}T^{j-1}\cdot diag\left( 1-a^{j-1} \right). \end{matrix} \left( S1-17 \right)$$

Here ${}_{n}U$ is the matrix with elements

$${}_{n}U_{x,r}=\left\{ \begin{matrix} 0 & r<x \\ \left( \prod_{x\leq k<r} \left( 1-m_{k} \right) \right)m_{r}/{{}_{n}m}_{x} & x\leq r<x+n \\ 0 & r\geq x+n \end{matrix} \right., \left( S1-18 \right)$$

Following [[2](#ref-dyrting2021)], conditional ratios are estimated by maximising $\mathcal{L}_{a}^{j}$ sequentially by iterated linear regressions. The regression remains the same as given in [[2](#ref-dyrting2021)] except that the expression for $G$ is generalized to

$$G=diag\left( \frac{1}{{}_{n}^{b}a\left( 1-{}_{n}^{b}a \right)} \right)\cdot{}_{n}^{b}T\cdot diag\left( a\left( 1-a \right) \right)\cdot B \left( S1-19 \right)$$

to account for age-grouping.

References

1. Dyrting S. Smoothing migration intensities with P-TOPALS. Demographic Research. 2020;43: 1607–1650. doi:[10.4054/DemRes.2020.43.55](https://doi.org/10.4054/DemRes.2020.43.55)

2. Dyrting S, Taylor A. Smoothing destination-specific migration flows. Annals of Regional Science. 2021;67: 359–383. doi:[10.1007/s00168-021-01051-4](https://doi.org/10.1007/s00168-021-01051-4)
